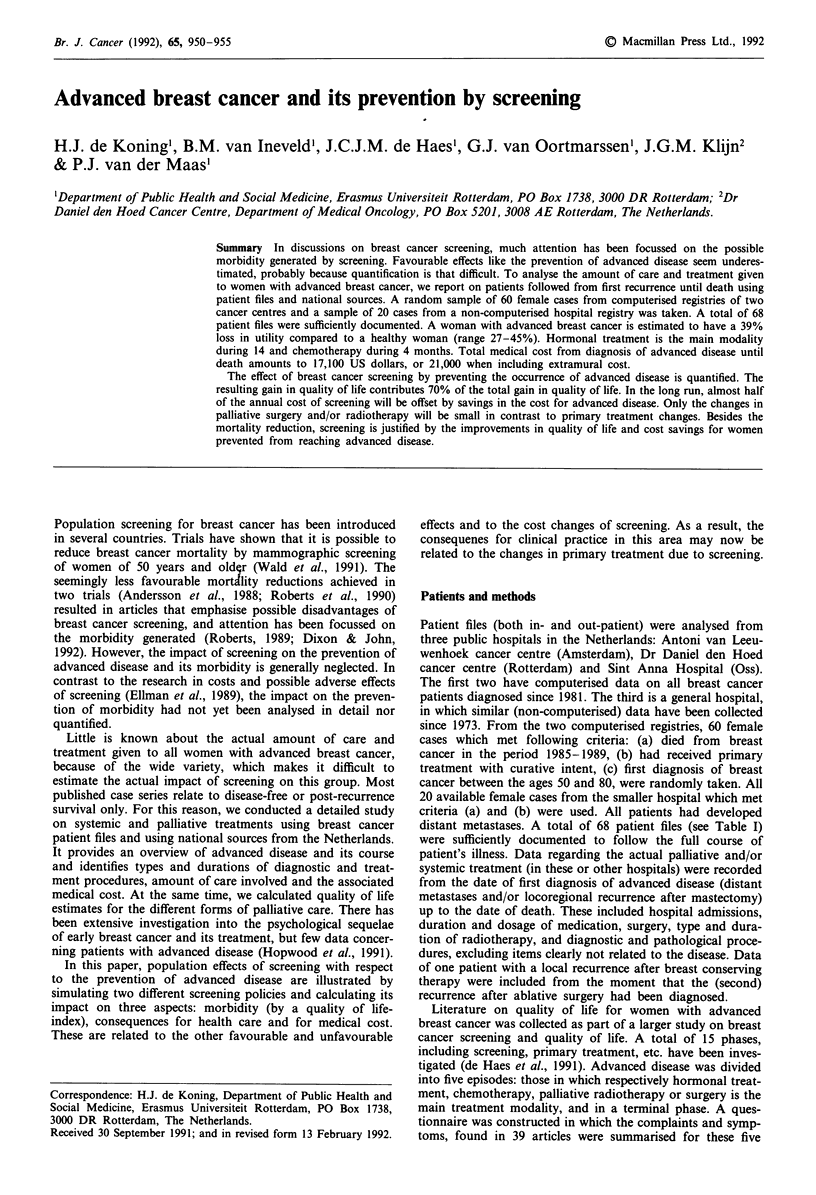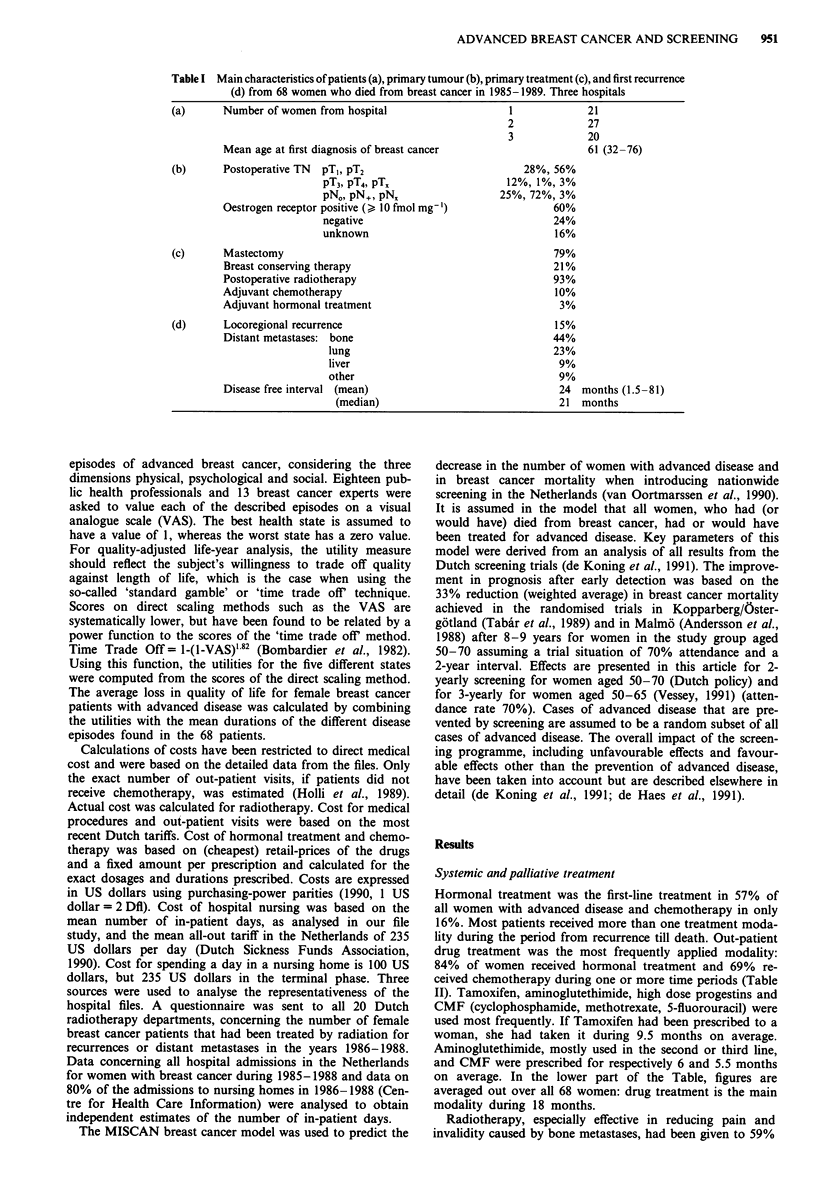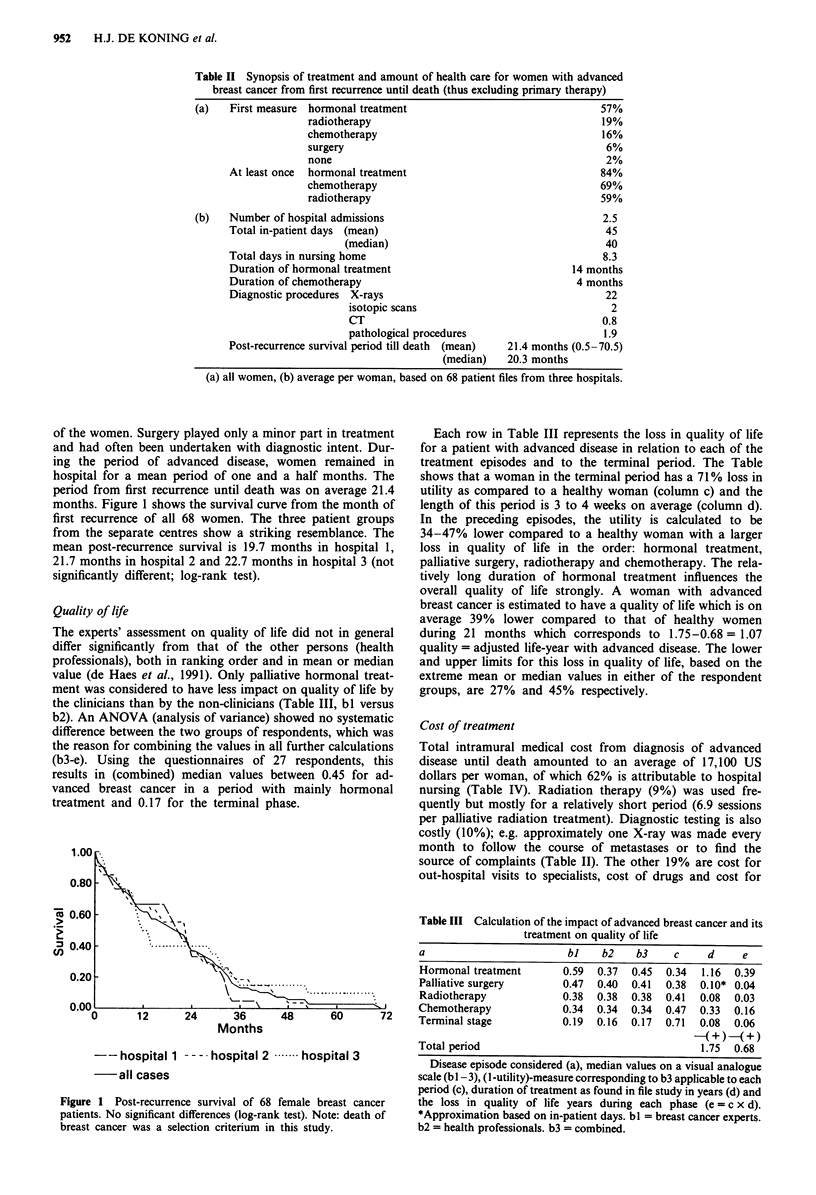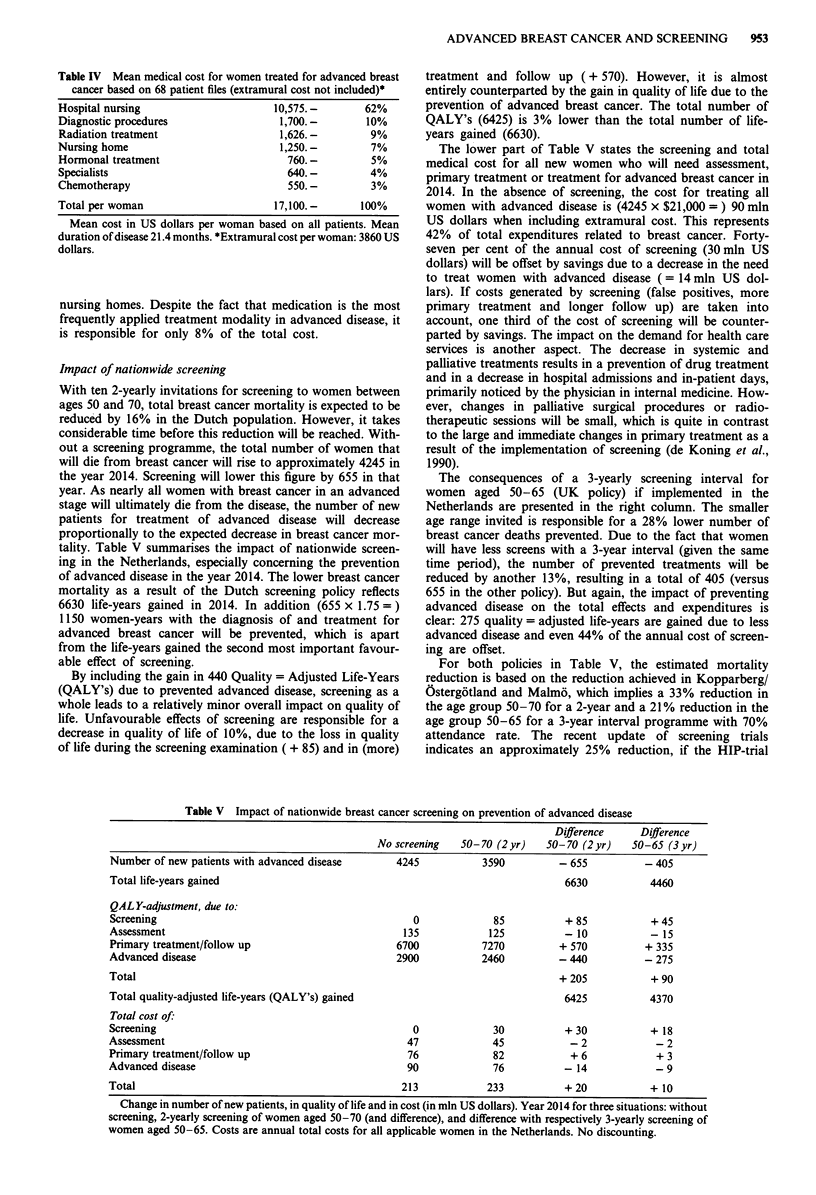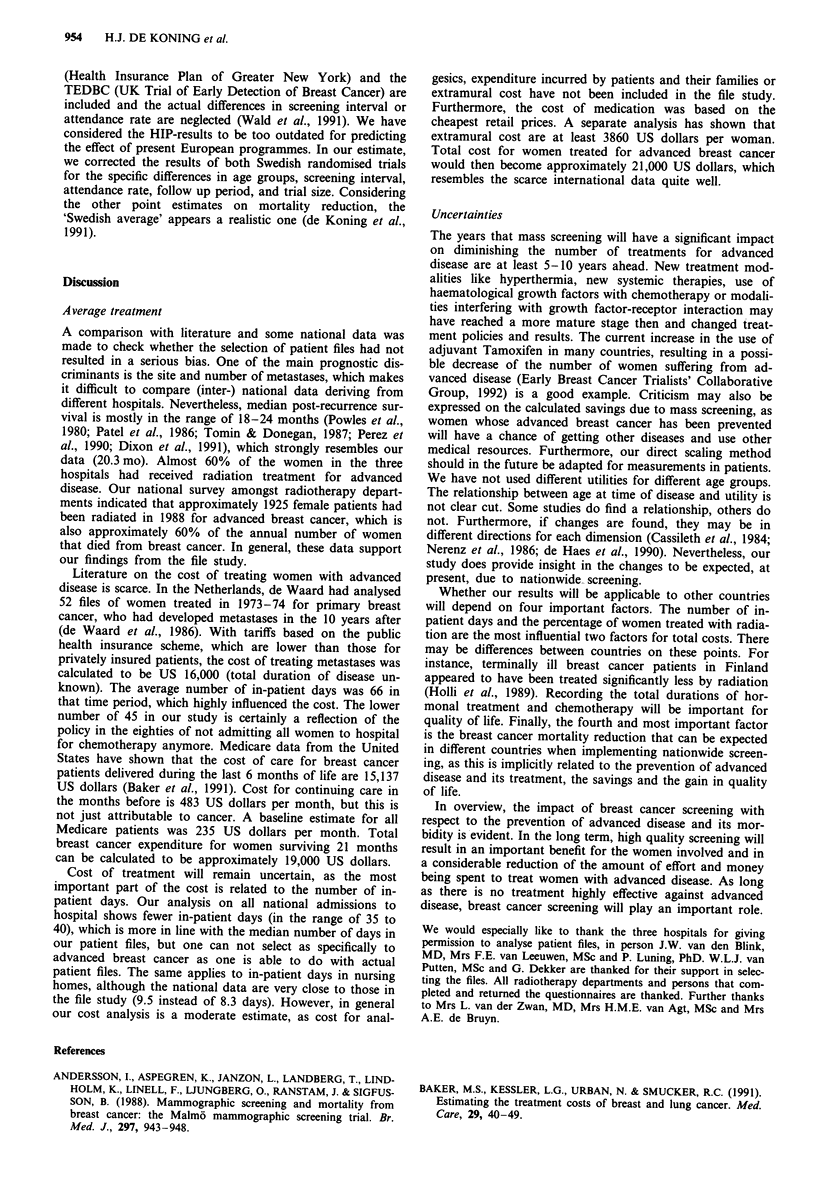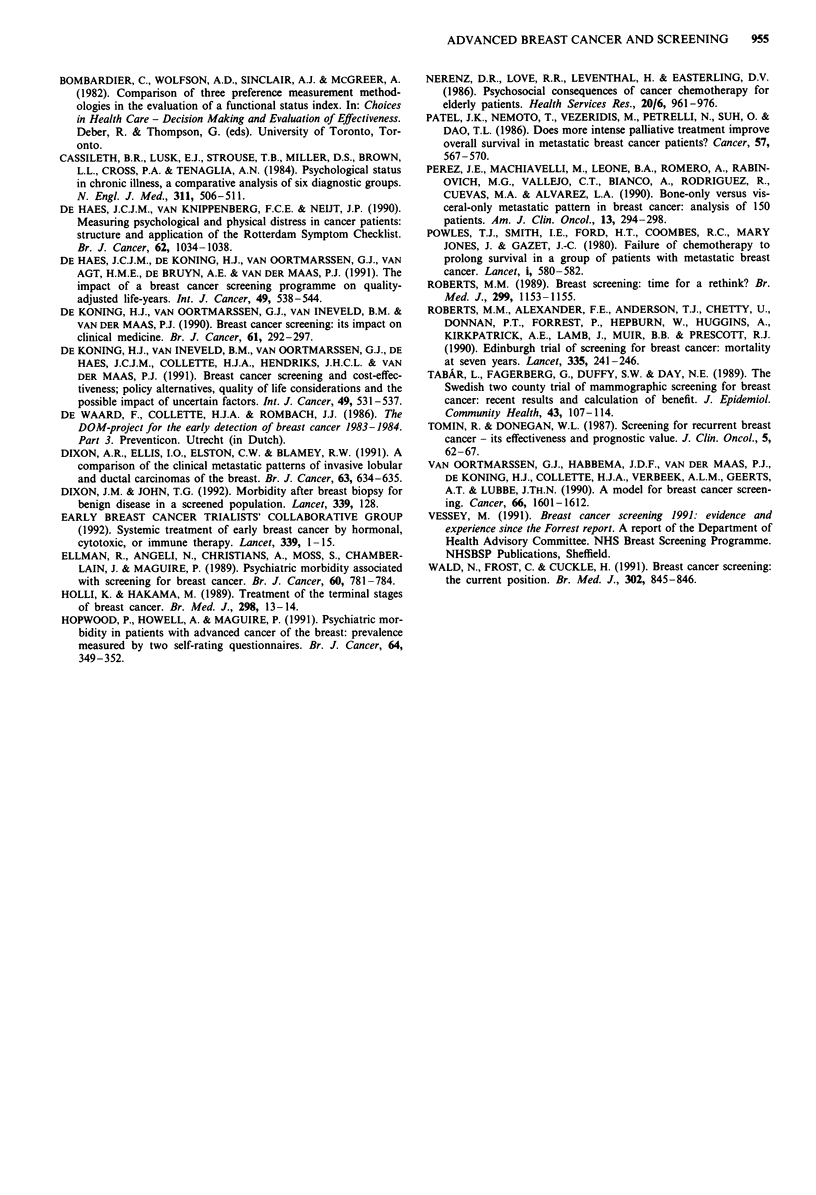# Advanced breast cancer and its prevention by screening.

**DOI:** 10.1038/bjc.1992.199

**Published:** 1992-06

**Authors:** H. J. de Koning, B. M. van Ineveld, J. C. de Haes, G. J. van Oortmarssen, J. G. Klijn, P. J. van der Maas

**Affiliations:** Department of Public Health and Social Medicine, Erasmus Universiteit Rotterdam, The Netherlands.

## Abstract

In discussions on breast cancer screening, much attention has been focussed on the possible morbidity generated by screening. Favourable effects like the prevention of advanced disease seem underestimated, probably because quantification is that difficult. To analyse the amount of care and treatment given to women with advanced breast cancer, we report on patients followed from first recurrence until death using patient files and national sources. A random sample of 60 female cases from computerised registries of two cancer centres and a sample of 20 cases from a non-computerised hospital registry was taken. A total of 68 patient files were sufficiently documented. A woman with advanced breast cancer is estimated to have a 39% loss in utility compared to a healthy woman (range 27-45%). Hormonal treatment is the main modality during 14 and chemotherapy during 4 months. Total medical cost from diagnosis of advanced disease until death amounts to 17,100 US dollars, or 21,000 when including extramural cost. The effect of breast cancer screening by preventing the occurrence of advanced disease is quantified. The resulting gain in quality of life contributes 70% of the total gain in quality of life. In the long run, almost half of the annual cost of screening will be offset by savings in the cost for advanced disease. Only the changes in palliative surgery and/or radiotherapy will be small in contrast to primary treatment changes. Besides the mortality reduction, screening is justified by the improvements in quality of life and cost savings for women prevented from reaching advanced disease.


Br. J. Cancer (1992), 65, 950-955
© Macmillan Press Ltd., 1992

### Advanced breast cancer and its prevention by screening

H.J. de Koning[1], B.M. van Ineveld[1], J.C.J.M. de Haes[1], G.J. van Oortmarssen[1], J.G.M. Klijn[2] & P.J. van der Maas[1]

[1]*Department of Public Health and Social Medicine, Erasmus Universiteit Rotterdam, PO Box 1738, 3000 DR Rotterdam;* [2]*Dr Daniel den Hoed Cancer Centre, Department of Medical Oncology, PO Box 5201, 3008 AE Rotterdam, The Netherlands.*

**Summary** In discussions on breast cancer screening, much attention has been focussed on the possible morbidity generated by screening. Favourable effects like the prevention of advanced disease seem underestimated, probably because quantification is that difficult. To analyse the amount of care and treatment given to women with advanced breast cancer, we report on patients followed from first recurrence until death using patient files and national sources. A random sample of 60 female cases from computerised registries of two cancer centres and a sample of 20 cases from a non-computerised hospital registry was taken. A total of 68 patient files were sufficiently documented. A woman with advanced breast cancer is estimated to have a 39% loss in utility compared to a healthy woman (range 27–45%). Hormonal treatment is the main modality during 14 and chemotherapy during 4 months. Total medical cost from diagnosis of advanced disease until death amounts to 17,100 US dollars, or 21,000 when including extramural cost.

The effect of breast cancer screening by preventing the occurrence of advanced disease is quantified. The resulting gain in quality of life contributes 70% of the total gain in quality of life. In the long run, almost half of the annual cost of screening will be offset by savings in the cost for advanced disease. Only the changes in palliative surgery and/or radiotherapy will be small in contrast to primary treatment changes. Besides the mortality reduction, screening is justified by the improvements in quality of life and cost savings for women prevented from reaching advanced disease.

Population screening for breast cancer has been introduced in several countries. Trials have shown that it is possible to reduce breast cancer mortality by mammographic screening of women of 50 years and older (Wald *et al.*, 1991). The seemingly less favourable mortality reductions achieved in two trials (Andersson *et al.*, 1988; Roberts *et al.*, 1990) resulted in articles that emphasise possible disadvantages of breast cancer screening, and attention has been focussed on the morbidity generated (Roberts, 1989; Dixon & John, 1992). However, the impact of screening on the prevention of advanced disease and its morbidity is generally neglected. In contrast to the research in costs and possible adverse effects of screening (Ellman *et al.*, 1989), the impact on the prevention of morbidity had not yet been analysed in detail nor quantified.

Little is known about the actual amount of care and treatment given to all women with advanced breast cancer, because of the wide variety, which makes it difficult to estimate the actual impact of screening on this group. Most published case series relate to disease-free or post-recurrence survival only. For this reason, we conducted a detailed study on systemic and palliative treatments using breast cancer patient files and using national sources from the Netherlands. It provides an overview of advanced disease and its course and identifies types and durations of diagnostic and treatment procedures, amount of care involved and the associated medical cost. At the same time, we calculated quality of life estimates for the different forms of palliative care. There has been extensive investigation into the psychological sequelae of early breast cancer and its treatment, but few data concerning patients with advanced disease (Hopwood *et al.*, 1991).

In this paper, population effects of screening with respect to the prevention of advanced disease are illustrated by simulating two different screening policies and calculating its impact on three aspects: morbidity (by a quality of life-index), consequences for health care and for medical cost. These are related to the other favourable and unfavourable effects and to the cost changes of screening. As a result, the consequenes for clinical practice in this area may now be related to the changes in primary treatment due to screening.

### Patients and methods

Patient files (both in- and out-patient) were analysed from three public hospitals in the Netherlands: Antoni van Leeuwenhoek cancer centre (Amsterdam), Dr Daniel den Hoed cancer centre (Rotterdam) and Sint Anna Hospital (Oss). The first two have computerised data on all breast cancer patients diagnosed since 1981. The third is a general hospital, in which similar (non-computerised) data have been collected since 1973. From the two computerised registries, 60 female cases which met following criteria: (a) died from breast cancer in the period 1985–1989, (b) had received primary treatment with curative intent, (c) first diagnosis of breast cancer between the ages 50 and 80, were randomly taken. All 20 available female cases from the smaller hospital which met criteria (a) and (b) were used. All patients had developed distant metastases. A total of 68 patient files (see Table I) were sufficiently documented to follow the full course of patient's illness. Data regarding the actual palliative and/or systemic treatment (in these or other hospitals) were recorded from the date of first diagnosis of advanced disease (distant metastases and/or locoregional recurrence after mastectomy) up to the date of death. These included hospital admissions, duration and dosage of medication, surgery, type and duration of radiotherapy, and diagnostic and pathological procedures, excluding items clearly not related to the disease. Data of one patient with a local recurrence after breast conserving therapy were included from the moment that the (second) recurrence after ablative surgery had been diagnosed.

Literature on quality of life for women with advanced breast cancer was collected as part of a larger study on breast cancer screening and quality of life. A total of 15 phases, including screening, primary treatment, etc. have been investigated (de Haes *et al.*, 1991). Advanced disease was divided into five episodes: those in which respectively hormonal treatment, chemotherapy, palliative radiotherapy or surgery is the main treatment modality, and in a terminal phase. A questionnaire was constructed in which the complaints and symptoms, found in 39 articles were summarised for these five

Correspondence: H.J. de Koning, Department of Public Health and Social Medicine, Erasmus Universiteit Rotterdam, PO Box 1738, 3000 DR Rotterdam, The Netherlands.
Received 30 September 1991; and in revised form 13 February 1992.

**Table I** Main characteristics of patients (a), primary tumour (b), primary treatment (c), and first recurrence (d) from 68 women who died from breast cancer in 1985–1989. Three hospitals

| | | | |
|---|---|---|---|
| (a) | Number of women from hospital | 1 | 21 |
| | | 2 | 27 |
| | | 3 | 20 |
| | Mean age at first diagnosis of breast cancer | | 61 (32–76) |
| (b) | Postoperative TN $pT_1$, $pT_2$ | 28%, 56% | |
| | $pT_3$, $pT_4$, $pT_x$ | 12%, 1%, 3% | |
| | $pN_0$, $pN_+$, $pN_x$ | 25%, 72%, 3% | |
| | Oestrogen receptor positive ($\geqslant$ 10 fmol $mg^{-1}$) | 60% | |
| | negative | 24% | |
| | unknown | 16% | |
| (c) | Mastectomy | 79% | |
| | Breast conserving therapy | 21% | |
| | Postoperative radiotherapy | 93% | |
| | Adjuvant chemotherapy | 10% | |
| | Adjuvant hormonal treatment | 3% | |
| (d) | Locoregional recurrence | 15% | |
| | Distant metastases: bone | 44% | |
| | lung | 23% | |
| | liver | 9% | |
| | other | 9% | |
| | Disease free interval (mean) | 24 | months (1.5–81) |
| | (median) | 21 | months |

episodes of advanced breast cancer, considering the three dimensions physical, psychological and social. Eighteen public health professionals and 13 breast cancer experts were asked to value each of the described episodes on a visual analogue scale (VAS). The best health state is assumed to have a value of 1, whereas the worst state has a zero value. For quality-adjusted life-year analysis, the utility measure should reflect the subject's willingness to trade off quality against length of life, which is the case when using the so-called 'standard gamble' or 'time trade off' technique. Scores on direct scaling methods such as the VAS are systematically lower, but have been found to be related by a power function to the scores of the 'time trade off' method. Time Trade Off = $1-(1-\text{VAS})^{1.82}$ (Bombardier *et al.*, 1982). Using this function, the utilities for the five different states were computed from the scores of the direct scaling method. The average loss in quality of life for female breast cancer patients with advanced disease was calculated by combining the utilities with the mean durations of the different disease episodes found in the 68 patients.

Calculations of costs have been restricted to direct medical cost and were based on the detailed data from the files. Only the exact number of out-patient visits, if patients did not receive chemotherapy, was estimated (Holli *et al.*, 1989). Actual cost was calculated for radiotherapy. Cost for medical procedures and out-patient visits were based on the most recent Dutch tariffs. Cost of hormonal treatment and chemotherapy was based on (cheapest) retail-prices of the drugs and a fixed amount per prescription and calculated for the exact dosages and durations prescribed. Costs are expressed in US dollars using purchasing-power parities (1990, 1 US dollar = 2 Dfl). Cost of hospital nursing was based on the mean number of in-patient days, as analysed in our file study, and the mean all-out tariff in the Netherlands of 235 US dollars per day (Dutch Sickness Funds Association, 1990). Cost for spending a day in a nursing home is 100 US dollars, but 235 US dollars in the terminal phase. Three sources were used to analyse the representativeness of the hospital files. A questionnaire was sent to all 20 Dutch radiotherapy departments, concerning the number of female breast cancer patients that had been treated by radiation for recurrences or distant metastases in the years 1986–1988. Data concerning all hospital admissions in the Netherlands for women with breast cancer during 1985–1988 and data on 80% of the admissions to nursing homes in 1986–1988 (Centre for Health Care Information) were analysed to obtain independent estimates of the number of in-patient days.

The MISCAN breast cancer model was used to predict the decrease in the number of women with advanced disease and in breast cancer mortality when introducing nationwide screening in the Netherlands (van Oortmarssen *et al.*, 1990). It is assumed in the model that all women, who had (or would have) died from breast cancer, had or would have been treated for advanced disease. Key parameters of this model were derived from an analysis of all results from the Dutch screening trials (de Koning *et al.*, 1991). The improvement in prognosis after early detection was based on the 33% reduction (weighted average) in breast cancer mortality achieved in the randomised trials in Kopparberg/Östergötland (Tabár *et al.*, 1989) and in Malmö (Andersson *et al.*, 1988) after 8–9 years for women in the study group aged 50–70 assuming a trial situation of 70% attendance and a 2-year interval. Effects are presented in this article for 2-yearly screening for women aged 50–70 (Dutch policy) and for 3-yearly for women aged 50–65 (Vessey, 1991) (attendance rate 70%). Cases of advanced disease that are prevented by screening are assumed to be a random subset of all cases of advanced disease. The overall impact of the screening programme, including unfavourable effects and favourable effects other than the prevention of advanced disease, have been taken into account but are described elsewhere in detail (de Koning *et al.*, 1991; de Haes *et al.*, 1991).

### Results

### *Systemic and palliative treatment*

Hormonal treatment was the first-line treatment in 57% of all women with advanced disease and chemotherapy in only 16%. Most patients received more than one treatment modality during the period from recurrence till death. Out-patient drug treatment was the most frequently applied modality: 84% of women received hormonal treatment and 69% received chemotherapy during one or more time periods (Table II). Tamoxifen, aminoglutethimide, high dose progestins and CMF (cyclophosphamide, methotrexate, 5-fluorouracil) were used most frequently. If Tamoxifen had been prescribed to a woman, she had taken it during 9.5 months on average. Aminoglutethimide, mostly used in the second or third line, and CMF were prescribed for respectively 6 and 5.5 months on average. In the lower part of the Table, figures are averaged out over all 68 women: drug treatment is the main modality during 18 months.

Radiotherapy, especially effective in reducing pain and invalidity caused by bone metastases, had been given to 59%

**Table II** Synopsis of treatment and amount of health care for women with advanced breast cancer from first recurrence until death (thus excluding primary therapy)

| | | | |
|---|---|---|---|
| (a) | First measure | hormonal treatment | 57% |
| | | radiotherapy | 19% |
| | | chemotherapy | 16% |
| | | surgery | 6% |
| | | none | 2% |
| | At least once | hormonal treatment | 84% |
| | | chemotherapy | 69% |
| | | radiotherapy | 59% |
| (b) | Number of hospital admissions | | 2.5 |
| | Total in-patient days | (mean) | 45 |
| | | (median) | 40 |
| | Total days in nursing home | | 8.3 |
| | Duration of hormonal treatment | | 14 months |
| | Duration of chemotherapy | | 4 months |
| | Diagnostic procedures | X-rays | 22 |
| | | isotopic scans | 2 |
| | | CT | 0.8 |
| | | pathological procedures | 1.9 |
| | Post-recurrence survival period till death | (mean) | 21.4 months (0.5–70.5) |
| | | (median) | 20.3 months |

(a) all women, (b) average per woman, based on 68 patient files from three hospitals.

of the women. Surgery played only a minor part in treatment and had often been undertaken with diagnostic intent. During the period of advanced disease, women remained in hospital for a mean period of one and a half months. The period from first recurrence until death was on average 21.4 months. Figure 1 shows the survival curve from the month of first recurrence of all 68 women. The three patient groups from the separate centres show a striking resemblance. The mean post-recurrence survival is 19.7 months in hospital 1, 21.7 months in hospital 2 and 22.7 months in hospital 3 (not significantly different; log-rank test).

*Quality of life*

The experts' assessment on quality of life did not in general differ significantly from that of the other persons (health professionals), both in ranking order and in mean or median value (de Haes *et al.*, 1991). Only palliative hormonal treatment was considered to have less impact on quality of life by the clinicians than by the non-clinicians (Table III, b1 versus b2). An ANOVA (analysis of variance) showed no systematic difference between the two groups of respondents, which was the reason for combining the values in all further calculations (b3-e). Using the questionnaires of 27 respondents, this results in (combined) median values between 0.45 for advanced breast cancer in a period with mainly hormonal treatment and 0.17 for the terminal phase.

**Figure 1** Post-recurrence survival of 68 female breast cancer patients. No significant differences (log-rank test). Note: death of breast cancer was a selection criterium in this study.

Each row in Table III represents the loss in quality of life for a patient with advanced disease in relation to each of the treatment episodes and to the terminal period. The Table shows that a woman in the terminal period has a 71% loss in utility as compared to a healthy woman (column c) and the length of this period is 3 to 4 weeks on average (column d). In the preceding episodes, the utility is calculated to be 34–47% lower compared to a healthy woman with a larger loss in quality of life in the order: hormonal treatment, palliative surgery, radiotherapy and chemotherapy. The relatively long duration of hormonal treatment influences the overall quality of life strongly. A woman with advanced breast cancer is estimated to have a quality of life which is on average 39% lower compared to that of healthy women during 21 months which corresponds to 1.75–0.68 = 1.07 quality = adjusted life-year with advanced disease. The lower and upper limits for this loss in quality of life, based on the extreme mean or median values in either of the respondent groups, are 27% and 45% respectively.

*Cost of treatment*

Total intramural medical cost from diagnosis of advanced disease until death amounted to an average of 17,100 US dollars per woman, of which 62% is attributable to hospital nursing (Table IV). Radiation therapy (9%) was used frequently but mostly for a relatively short period (6.9 sessions per palliative radiation treatment). Diagnostic testing is also costly (10%); e.g. approximately one X-ray was made every month to follow the course of metastases or to find the source of complaints (Table II). The other 19% are cost for out-hospital visits to specialists, cost of drugs and cost for

**Table III** Calculation of the impact of advanced breast cancer and its treatment on quality of life

| *a* | *b1* | *b2* | *b3* | *c* | *d* | *e* |
|---|---|---|---|---|---|---|
| Hormonal treatment | 0.59 | 0.37 | 0.45 | 0.34 | 1.16 | 0.39 |
| Palliative surgery | 0.47 | 0.40 | 0.41 | 0.38 | 0.10* | 0.04 |
| Radiotherapy | 0.38 | 0.38 | 0.38 | 0.41 | 0.08 | 0.03 |
| Chemotherapy | 0.34 | 0.34 | 0.34 | 0.47 | 0.33 | 0.16 |
| Terminal stage | 0.19 | 0.16 | 0.17 | 0.71 | 0.08 | 0.06 |
| | | | | | —(+) | —(+) |
| Total period | | | | | 1.75 | 0.68 |

Disease episode considered (a), median values on a visual analogue scale (b1–3), (1-utility)-measure corresponding to b3 applicable to each period (c), duration of treatment as found in file study in years (d) and the loss in quality of life years during each phase (e = c × d). *Approximation based on in-patient days. b1 = breast cancer experts. b2 = health professionals. b3 = combined.

**Table IV** Mean medical cost for women treated for advanced breast cancer based on 68 patient files (extramural cost not included)*

| | | |
|---|---|---|
| Hospital nursing | 10,575. – | 62% |
| Diagnostic procedures | 1,700. – | 10% |
| Radiation treatment | 1,626. – | 9% |
| Nursing home | 1,250. – | 7% |
| Hormonal treatment | 760. – | 5% |
| Specialists | 640. – | 4% |
| Chemotherapy | 550. – | 3% |
| Total per woman | 17,100. – | 100% |

Mean cost in US dollars per woman based on all patients. Mean duration of disease 21.4 months. *Extramural cost per woman: 3860 US dollars.

nursing homes. Despite the fact that medication is the most frequently applied treatment modality in advanced disease, it is responsible for only 8% of the total cost.

### *Impact of nationwide screening*

With ten 2-yearly invitations for screening to women between ages 50 and 70, total breast cancer mortality is expected to be reduced by 16% in the Dutch population. However, it takes considerable time before this reduction will be reached. Without a screening programme, the total number of women that will die from breast cancer will rise to approximately 4245 in the year 2014. Screening will lower this figure by 655 in that year. As nearly all women with breast cancer in an advanced stage will ultimately die from the disease, the number of new patients for treatment of advanced disease will decrease proportionally to the expected decrease in breast cancer mortality. Table V summarises the impact of nationwide screening in the Netherlands, especially concerning the prevention of advanced disease in the year 2014. The lower breast cancer mortality as a result of the Dutch screening policy reflects 6630 life-years gained in 2014. In addition (655 × 1.75 = ) 1150 women-years with the diagnosis of and treatment for advanced breast cancer will be prevented, which is apart from the life-years gained the second most important favourable effect of screening.

By including the gain in 440 Quality = Adjusted Life-Years (QALY's) due to prevented advanced disease, screening as a whole leads to a relatively minor overall impact on quality of life. Unfavourable effects of screening are responsible for a decrease in quality of life of 10%, due to the loss in quality of life during the screening examination ( + 85) and in (more) treatment and follow up ( + 570). However, it is almost entirely counterparted by the gain in quality of life due to the prevention of advanced breast cancer. The total number of QALY's (6425) is 3% lower than the total number of life-years gained (6630).

The lower part of Table V states the screening and total medical cost for all new women who will need assessment, primary treatment or treatment for advanced breast cancer in 2014. In the absence of screening, the cost for treating all women with advanced disease is (4245 × $21,000 = ) 90 mln US dollars when including extramural cost. This represents 42% of total expenditures related to breast cancer. Forty-seven per cent of the annual cost of screening (30 mln US dollars) will be offset by savings due to a decrease in the need to treat women with advanced disease ( = 14 mln US dollars). If costs generated by screening (false positives, more primary treatment and longer follow up) are taken into account, one third of the cost of screening will be counterparted by savings. The impact on the demand for health care services is another aspect. The decrease in systemic and palliative treatments results in a prevention of drug treatment and in a decrease in hospital admissions and in-patient days, primarily noticed by the physician in internal medicine. However, changes in palliative surgical procedures or radiotherapeutic sessions will be small, which is quite in contrast to the large and immediate changes in primary treatment as a result of the implementation of screening (de Koning *et al.*, 1990).

The consequences of a 3-yearly screening interval for women aged 50–65 (UK policy) if implemented in the Netherlands are presented in the right column. The smaller age range invited is responsible for a 28% lower number of breast cancer deaths prevented. Due to the fact that women will have less screens with a 3-year interval (given the same time period), the number of prevented treatments will be reduced by another 13%, resulting in a total of 405 (versus 655 in the other policy). But again, the impact of preventing advanced disease on the total effects and expenditures is clear: 275 quality = adjusted life-years are gained due to less advanced disease and even 44% of the annual cost of screening are offset.

For both policies in Table V, the estimated mortality reduction is based on the reduction achieved in Kopparberg/Östergötland and Malmö, which implies a 33% reduction in the age group 50–70 for a 2-year and a 21% reduction in the age group 50–65 for a 3-year interval programme with 70% attendance rate. The recent update of screening trials indicates an approximately 25% reduction, if the HIP-trial

**Table V** Impact of nationwide breast cancer screening on prevention of advanced disease

| | *No screening* | *50–70 (2 yr)* | *Difference 50–70 (2 yr)* | *Difference 50–65 (3 yr)* |
|---|---|---|---|---|
| Number of new patients with advanced disease | 4245 | 3590 | – 655 | – 405 |
| Total life-years gained | | | 6630 | 4460 |
| *QALY-adjustment, due to:* | | | | |
| Screening | 0 | 85 | + 85 | + 45 |
| Assessment | 135 | 125 | – 10 | – 15 |
| Primary treatment/follow up | 6700 | 7270 | + 570 | + 335 |
| Advanced disease | 2900 | 2460 | – 440 | – 275 |
| Total | | | + 205 | + 90 |
| Total quality-adjusted life-years (QALY's) gained | | | 6425 | 4370 |
| *Total cost of:* | | | | |
| Screening | 0 | 30 | + 30 | + 18 |
| Assessment | 47 | 45 | – 2 | – 2 |
| Primary treatment/follow up | 76 | 82 | + 6 | + 3 |
| Advanced disease | 90 | 76 | – 14 | – 9 |
| Total | 213 | 233 | + 20 | + 10 |

Change in number of new patients, in quality of life and in cost (in mln US dollars). Year 2014 for three situations: without screening, 2-yearly screening of women aged 50–70 (and difference), and difference with respectively 3-yearly screening of women aged 50–65. Costs are annual total costs for all applicable women in the Netherlands. No discounting.

(Health Insurance Plan of Greater New York) and the TEDBC (UK Trial of Early Detection of Breast Cancer) are included and the actual differences in screening interval or attendance rate are neglected (Wald *et al.*, 1991). We have considered the HIP-results to be too outdated for predicting the effect of present European programmes. In our estimate, we corrected the results of both Swedish randomised trials for the specific differences in age groups, screening interval, attendance rate, follow up period, and trial size. Considering the other point estimates on mortality reduction, the 'Swedish average' appears a realistic one (de Koning *et al.*, 1991).

### Discussion

### *Average treatment*

A comparison with literature and some national data was made to check whether the selection of patient files had not resulted in a serious bias. One of the main prognostic discriminants is the site and number of metastases, which makes it difficult to compare (inter-) national data deriving from different hospitals. Nevertheless, median post-recurrence survival is mostly in the range of 18–24 months (Powles *et al.*, 1980; Patel *et al.*, 1986; Tomin & Donegan, 1987; Perez *et al.*, 1990; Dixon *et al.*, 1991), which strongly resembles our data (20.3 mo). Almost 60% of the women in the three hospitals had received radiation treatment for advanced disease. Our national survey amongst radiotherapy departments indicated that approximately 1925 female patients had been radiated in 1988 for advanced breast cancer, which is also approximately 60% of the annual number of women that died from breast cancer. In general, these data support our findings from the file study.

Literature on the cost of treating women with advanced disease is scarce. In the Netherlands, de Waard had analysed 52 files of women treated in 1973–74 for primary breast cancer, who had developed metastases in the 10 years after (de Waard *et al.*, 1986). With tariffs based on the public health insurance scheme, which are lower than those for privately insured patients, the cost of treating metastases was calculated to be US 16,000 (total duration of disease unknown). The average number of in-patient days was 66 in that time period, which highly influenced the cost. The lower number of 45 in our study is certainly a reflection of the policy in the eighties of not admitting all women to hospital for chemotherapy anymore. Medicare data from the United States have shown that the cost of care for breast cancer patients delivered during the last 6 months of life are 15,137 US dollars (Baker *et al.*, 1991). Cost for continuing care in the months before is 483 US dollars per month, but this is not just attributable to cancer. A baseline estimate for all Medicare patients was 235 US dollars per month. Total breast cancer expenditure for women surviving 21 months can be calculated to be approximately 19,000 US dollars.

Cost of treatment will remain uncertain, as the most important part of the cost is related to the number of in-patient days. Our analysis on all national admissions to hospital shows fewer in-patient days (in the range of 35 to 40), which is more in line with the median number of days in our patient files, but one can not select as specifically to advanced breast cancer as one is able to do with actual patient files. The same applies to in-patient days in nursing homes, although the national data are very close to those in the file study (9.5 instead of 8.3 days). However, in general our cost analysis is a moderate estimate, as cost for analgesics, expenditure incurred by patients and their families or extramural cost have not been included in the file study. Furthermore, the cost of medication was based on the cheapest retail prices. A separate analysis has shown that extramural cost are at least 3860 US dollars per woman. Total cost for women treated for advanced breast cancer would then become approximately 21,000 US dollars, which resembles the scarce international data quite well.

### *Uncertainties*

The years that mass screening will have a significant impact on diminishing the number of treatments for advanced disease are at least 5–10 years ahead. New treatment modalities like hyperthermia, new systemic therapies, use of haematological growth factors with chemotherapy or modalities interfering with growth factor-receptor interaction may have reached a more mature stage then and changed treatment policies and results. The current increase in the use of adjuvant Tamoxifen in many countries, resulting in a possible decrease of the number of women suffering from advanced disease (Early Breast Cancer Trialists' Collaborative Group, 1992) is a good example. Criticism may also be expressed on the calculated savings due to mass screening, as women whose advanced breast cancer has been prevented will have a chance of getting other diseases and use other medical resources. Furthermore, our direct scaling method should in the future be adapted for measurements in patients. We have not used different utilities for different age groups. The relationship between age at time of disease and utility is not clear cut. Some studies do find a relationship, others do not. Furthermore, if changes are found, they may be in different directions for each dimension (Cassileth *et al.*, 1984; Nerenz *et al.*, 1986; de Haes *et al.*, 1990). Nevertheless, our study does provide insight in the changes to be expected, at present, due to nationwide screening.

Whether our results will be applicable to other countries will depend on four important factors. The number of in-patient days and the percentage of women treated with radiation are the most influential two factors for total costs. There may be differences between countries on these points. For instance, terminally ill breast cancer patients in Finland appeared to have been treated significantly less by radiation (Holli *et al.*, 1989). Recording the total durations of hormonal treatment and chemotherapy will be important for quality of life. Finally, the fourth and most important factor is the breast cancer mortality reduction that can be expected in different countries when implementing nationwide screening, as this is implicitly related to the prevention of advanced disease and its treatment, the savings and the gain in quality of life.

In overview, the impact of breast cancer screening with respect to the prevention of advanced disease and its morbidity is evident. In the long term, high quality screening will result in an important benefit for the women involved and in a considerable reduction of the amount of effort and money being spent to treat women with advanced disease. As long as there is no treatment highly effective against advanced disease, breast cancer screening will play an important role.

We would especially like to thank the three hospitals for giving permission to analyse patient files, in person J.W. van den Blink, MD, Mrs F.E. van Leeuwen, MSc and P. Luning, PhD. W.L.J. van Putten, MSc and G. Dekker are thanked for their support in selecting the files. All radiotherapy departments and persons that completed and returned the questionnaires are thanked. Further thanks to Mrs L. van der Zwan, MD, Mrs H.M.E. van Agt, MSc and Mrs A.E. de Bruyn.

### References

ANDERSSON, I., ASPEGREN, K., JANZON, L., LANDBERG, T., LINDHOLM, K., LINELL, F., LJUNGBERG, O., RANSTAM, J. & SIGFUSSON, B. (1988). Mammographic screening and mortality from breast cancer: the Malmö mammographic screening trial. *Br. Med. J.*, **297**, 943–948.

BAKER, M.S., KESSLER, L.G., URBAN, N. & SMUCKER, R.C. (1991). Estimating the treatment costs of breast and lung cancer. *Med. Care*, **29**, 40–49.

BOMBARDIER, C., WOLFSON, A.D., SINCLAIR, A.J. & MCGREER, A. (1982). Comparison of three preference measurement methodologies in the evaluation of a functional status index. In: *Choices in Health Care – Decision Making and Evaluation of Effectiveness*. Deber, R. & Thompson, G. (eds). University of Toronto, Toronto.

CASSILETH, B.R., LUSK, E.J., STROUSE, T.B., MILLER, D.S., BROWN, L.L., CROSS, P.A. & TENAGLIA, A.N. (1984). Psychological status in chronic illness, a comparative analysis of six diagnostic groups. *N. Engl. J. Med.*, **311**, 506–511.

DE HAES, J.C.J.M., VAN KNIPPENBERG, F.C.E. & NEIJT, J.P. (1990). Measuring psychological and physical distress in cancer patients: structure and application of the Rotterdam Symptom Checklist. *Br. J. Cancer*, **62**, 1034–1038.

DE HAES, J.C.J.M., DE KONING, H.J., VAN OORTMARSSEN, G.J., VAN AGT, H.M.E., DE BRUYN, A.E. & VAN DER MAAS, P.J. (1991). The impact of a breast cancer screening programme on quality-adjusted life-years. *Int. J. Cancer*, **49**, 538–544.

DE KONING, H.J., VAN OORTMARSSEN, G.J., VAN INEVELD, B.M. & VAN DER MAAS, P.J. (1990). Breast cancer screening: its impact on clinical medicine. *Br. J. Cancer*, **61**, 292–297.

DE KONING, H.J., VAN INEVELD, B.M., VAN OORTMARSSEN, G.J., DE HAES, J.C.J.M., COLLETTE, H.J.A., HENDRIKS, J.H.C.L. & VAN DER MAAS, P.J. (1991). Breast cancer screening and cost-effectiveness; policy alternatives, quality of life considerations and the possible impact of uncertain factors. *Int. J. Cancer*, **49**, 531–537.

DE WAARD, F., COLLETTE, H.J.A. & ROMBACH, J.J. (1986). *The DOM-project for the early detection of breast cancer 1983–1984. Part 3.* Preventicon. Utrecht (in Dutch).

DIXON, A.R., ELLIS, I.O., ELSTON, C.W. & BLAMEY, R.W. (1991). A comparison of the clinical metastatic patterns of invasive lobular and ductal carcinomas of the breast. *Br. J. Cancer*, **63**, 634–635.

DIXON, J.M. & JOHN, T.G. (1992). Morbidity after breast biopsy for benign disease in a screened population. *Lancet*, **339**, 128.

EARLY BREAST CANCER TRIALISTS' COLLABORATIVE GROUP (1992). Systemic treatment of early breast cancer by hormonal, cytotoxic, or immune therapy. *Lancet*, **339**, 1–15.

ELLMAN, R., ANGELI, N., CHRISTIANS, A., MOSS, S., CHAMBERLAIN, J. & MAGUIRE, P. (1989). Psychiatric morbidity associated with screening for breast cancer. *Br. J. Cancer*, **60**, 781–784.

HOLLI, K. & HAKAMA, M. (1989). Treatment of the terminal stages of breast cancer. *Br. Med. J.*, **298**, 13–14.

HOPWOOD, P., HOWELL, A. & MAGUIRE, P. (1991). Psychiatric morbidity in patients with advanced cancer of the breast: prevalence measured by two self-rating questionnaires. *Br. J. Cancer*, **64**, 349–352.

NERENZ, D.R., LOVE, R.R., LEVENTHAL, H. & EASTERLING, D.V. (1986). Psychosocial consequences of cancer chemotherapy for elderly patients. *Health Services Res.*, **20/6**, 961–976.

PATEL, J.K., NEMOTO, T., VEZERIDIS, M., PETRELLI, N., SUH, O. & DAO, T.L. (1986). Does more intense palliative treatment improve overall survival in metastatic breast cancer patients? *Cancer*, **57**, 567–570.

PEREZ, J.E., MACHIAVELLI, M., LEONE, B.A., ROMERO, A., RABINOVICH, M.G., VALLEJO, C.T., BIANCO, A., RODRIGUEZ, R., CUEVAS, M.A. & ALVAREZ, L.A. (1990). Bone-only versus visceral-only metastatic pattern in breast cancer: analysis of 150 patients. *Am. J. Clin. Oncol.*, **13**, 294–298.

POWLES, T.J., SMITH, I.E., FORD, H.T., COOMBES, R.C., MARY JONES, J. & GAZET, J.-C. (1980). Failure of chemotherapy to prolong survival in a group of patients with metastatic breast cancer. *Lancet*, **i**, 580–582.

ROBERTS, M.M. (1989). Breast screening: time for a rethink? *Br. Med. J.*, **299**, 1153–1155.

ROBERTS, M.M., ALEXANDER, F.E., ANDERSON, T.J., CHETTY, U., DONNAN, P.T., FORREST, P., HEPBURN, W., HUGGINS, A., KIRKPATRICK, A.E., LAMB, J., MUIR, B.B. & PRESCOTT, R.J. (1990). Edinburgh trial of screening for breast cancer: mortality at seven years. *Lancet*, **335**, 241–246.

TABÁR, L., FAGERBERG, G., DUFFY, S.W. & DAY, N.E. (1989). The Swedish two county trial of mammographic screening for breast cancer: recent results and calculation of benefit. *J. Epidemiol. Community Health*, **43**, 107–114.

TOMIN, R. & DONEGAN, W.L. (1987). Screening for recurrent breast cancer – its effectiveness and prognostic value. *J. Clin. Oncol.*, **5**, 62–67.

VAN OORTMARSSEN, G.J., HABBEMA, J.D.F., VAN DER MAAS, P.J., DE KONING, H.J., COLLETTE, H.J.A., VERBEEK, A.L.M., GEERTS, A.T. & LUBBE, J.TH.N. (1990). A model for breast cancer screening. *Cancer*, **66**, 1601–1612.

VESSEY, M. (1991). *Breast cancer screening 1991: evidence and experience since the Forrest report.* A report of the Department of Health Advisory Committee. NHS Breast Screening Programme. NHSBSP Publications, Sheffield.

WALD, N., FROST, C. & CUCKLE, H. (1991). Breast cancer screening: the current position. *Br. Med. J.*, **302**, 845–846.